# Interventional treatment of traumatic carotid-cavernous fistula: A case report

**DOI:** 10.1097/MD.0000000000032265

**Published:** 2022-12-30

**Authors:** Jiabin Wang, Xiaoming Shen, Niu Miao, Guofang Yang, Mingqin Zhang, Dongyi Yang, Yangyang Liu, Tao Wu

**Affiliations:** a Department of Intervention, The First Affiliated Hospital of Henan University of Traditional Chinese Medicine, Zhengzhou, Henan, China.

**Keywords:** cavernous fistula, covered stent, interventional treatment, traumatic carotid

## Abstract

Patients with traumatic carotid-cavernous fistula (TCCF) usually go to the ophthalmology department first because of the symptoms such as protrusion of eyes, edema and congestion of combined membrane, vision loss and so on. It is easy to be misdiagnosed and missed.

**Patient concerns:**

We report a case of left eye swelling and vision loss caused by TCCF after head injury due to traffic accident, which failed to respond to ophthalmic treatment for many times. The similar situation is very likely to cause panic among patients.

**Diagnosis:**

Cerebral angiography revealed left internal carotid-cavernous fistula (high flow type).

**Interventions:**

Left internal carotid artery covered stent implantation was performed.

**Outcomes:**

The fistulas and the original venous mass were completely covered by the covered stent, and the development of the vascular mass disappeared. The patient’s eye symptoms basically disappeared 14 days after the operation.

**Lessons:**

Interventional treatment of TCCF is effective.

## 1. Introduction

Traumatic carotid-cavernous fistula (TCCF) is a rare and special disease that causes the rupture of the arterial wall or its branches of the carotid cavernous sinus segment due to trauma, leading to the formation of abnormal arteriovenous communication between it and the cavernous sinus. The main clinical manifestations are pulsatile exophthalmos, conjunctival edema and congestion, nystagmus, vascular murmur and visual impairment immediately or several days and weeks after injury. In addition, symptoms such as cavernous sinus and supraorbital fissure syndrome may also occur.^[1]^ In clinical practice, TCCF patients often go to the ophthalmology department for exophthalmos, conjunctival edema, congestion, vision loss, etc. The vast majority of them has no obvious clinical effect, or even continues to worsen. They are often considered to be caused by this disease only after consulting the neurology department. It is very easy to misdiagnose and miss diagnosis in clinical practice, and must maintain a high degree of vigilance. This paper reports the clinical symptoms and imaging findings of a patient with left eye swelling and vision loss caused by TCCF after interventional treatment due to traumatic brain injury caused by a car accident.

## 2. Case presentation

**Figure 1.**
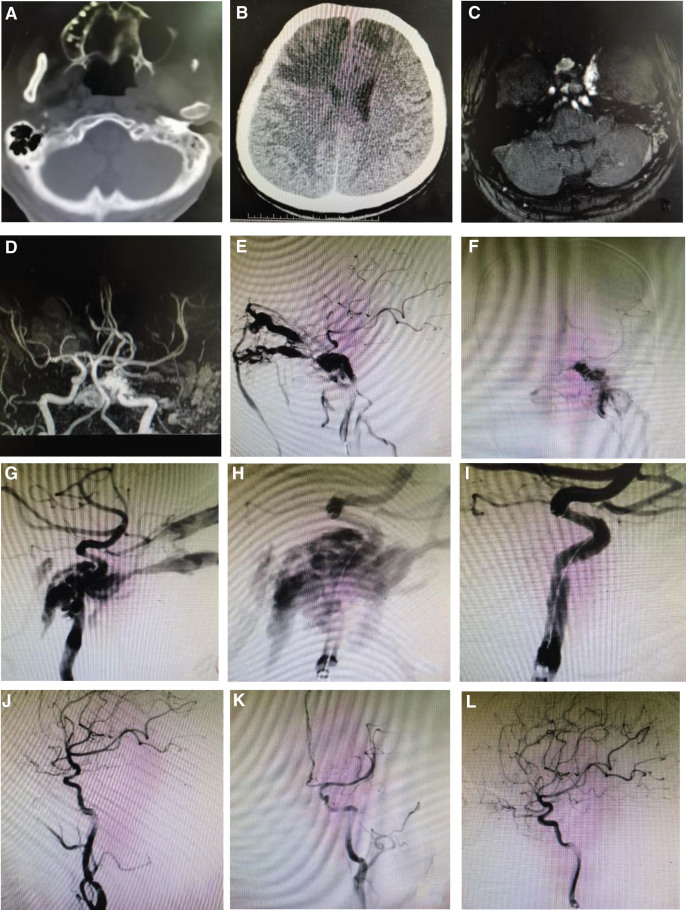
(A) Head CT shows nasal bone and occipital bone fracture. (B) Brain CT showed frontal lobe contusion and laceration. (C) Abnormal high signal intensity of cavernous sinus on MRI. (D) MRA showed more tortuous vascular shadows at the siphon of the left internal carotid artery. (E‐G) DSA showed that the left internal carotid artery cavernous sinus segment had a large amount of contrast agent infiltrating into the cavernous sinus, and the superior, inferior and peripheral venous vessels were significantly dilated. (H) Intraoperative angiography after placement of covered stent. (I‐K) Immediately after the release of the covered stent, angiography, lateral and anteroposterior angiography showed that the fistula was completely covered, and the original vein vascular mass was developed and disappeared. (L) Six months after operation, DSA showed that the fistula disappeared completely, did not recur, and the blood flow in the stent was smooth. CT = computed tomography, DSA = digital subtraction angiography, MRA = magnetic resonance arteriography, MRI = magnetic resonance imaging.

A 48-year-old male patient was first admitted to Puyang Oilfield General Hospital on the 17th day due to coma and irritability caused by car accident. Head computed tomography (CT) examination revealed occipital fracture, bilateral frontal lobe and left cerebellar hemisphere contusion with high density hematoma, left brachium pontine and pons with uneven reduced density contusion, subarachnoid hemorrhage, a small amount of right frontal subdural hemorrhage, and multiple nasal bone fractures on both sides. After the subdural hematoma was drilled and piped for drainage, and blood transfusion and symptomatic support treatment were given, the patient’s condition gradually improved. During the hospitalization, the patient was treated in the ophthalmology department for many times due to exophthalmos of the left eye, eyelid swelling, conjunctival congestion, edema, etc, but the symptoms continued to worsen. This time, the patient was hospitalized in our hospital for further diagnosis and treatment. Physical examination: clear consciousness, manic, left eye protrusion, eyelid swelling, conjunctival congestion, edema, blurred vision, left eye and temporal stethoscope can be heard and blowing blood vessel murmur, left eye ball examination cannot cooperate. Head CT examination after admission revealed nasal bone and occipital bone fractures, bilateral frontal lobe contusion and laceration (Fig. 1A and B). Head magnetic resonance imaging (MRI) + magnetic resonance arteriography (MRA) showed: bilateral frontotemporal parietal lobe, anterior longitudinal fissure cistern, left occipital subdural hemorrhage, subarachnoid hemorrhage, left eyeball protruding laterally, bilateral superior ophthalmic veins thickened (Fig. 1C and D). Traumatic cavernous sinus fistula was considered. Many tortuous vascular shadows were seen at the siphon of left internal carotid artery connected with it, and traumatic cavernous sinus fistula was considered. Previously, due to eye symptoms, many times of visits to the ophthalmology department did not improve, so please consult the interventional department. Digital subtraction angiography (DSA) showed that a large amount of contrast agent overflowed onto the eyes of the cavernous sinus of the left internal carotid artery, and the veins at the right and around were significantly dilated (Fig. 1E‐L). Diagnosis: left internal carotid artery cavernous sinus fistula (high flow type). The left internal carotid artery covered stent implantation will be performed on March 1.

On March 1, 2022, under combined intravenous and inhalation general anesthesia, a 6F guide catheter was placed in the left internal carotid artery petrous segment through the 6F long sheath, and a nerve microcatheter was placed through the supporting catheter. The angiogram showed that the fistula was located in the left internal carotid artery cavernous sinus segment. The contrast agent was rapidly developed through the vein, the upper and lower internal carotid arteries were well filled and developed, the tube wall was smooth, and the microcatheter guided by the micro guide wire was placed in the left middle cerebral artery. A nerve exchange microguide wire (ASAHI, 300 cm) is placed through a microcatheter. The intracranial supporting catheter is guided to the end of the internal carotid artery and the microcatheter is withdrawn. A stent (WILLIS, 4 * 13 mm) is placed through the exchange microguide wire. After the stent was released, the angiogram showed that the stent was well positioned, attached to the wall and expanded, completely covered the fistula, the original vein vascular mass developed and disappeared, no contrast agent overflowed and remained between the walls, the internal carotid artery was unobstructed and developed well, the angiogram of delayed observation showed that the original developing vein was not developed, the stent was well attached to the wall and expanded, the left middle cerebral artery was well filled and developed, and the catheters at all levels were withdrawn. There was no abnormal reaction during the operation. After recovery from anesthesia, the blow like vascular murmur disappeared in the left eye and temporal region, and there was no abnormality in nerve examination.

**Figure 2.**
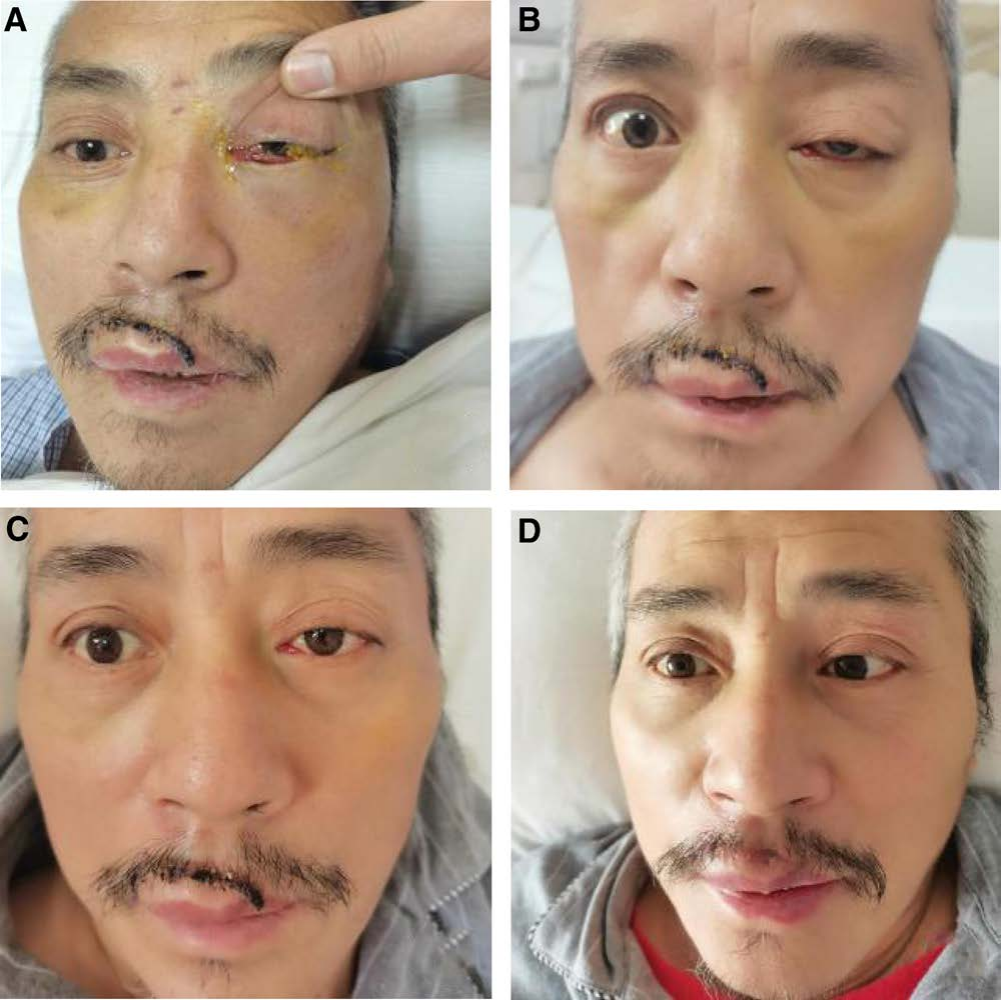
(A) Preoperative left eye exophthalmos, eyelid swelling, conjunctival congestion and edema. (B) On the third day after operation, the swelling, congestion and edema of the left eye were less than before. (C) The left eye improved significantly 7 days after operation. (D) The left eye returned to normal 14 days after operation.

After operation, low molecular weight heparin calcium injection (AOSIDA) 0.4 mL: 4100AXaIU was hypodermic injection (ih) for every 12 hours (Q12h). After 3 days of use, aspirin 100 mg peros (po), clopidogrel 75 mg po were prescribed. On the third day after operation, the left eyeball protrusion, eyelid swelling, conjunctival congestion, edema and blurred vision were significantly reduced (Fig. 2A and B). The left eye basically returned to normal on the 14th postoperative day (Fig. 2C and D). DSA review 6 months after surgery showed no abnormality.

## 3. Discussion

TCCF is a rare complication of head trauma with potentially serious consequences.^[2]^ About 0.3% of craniofacial trauma is associated with carotid cavernous sinus fistula, forming arteriovenous traffic from internal carotid artery or external carotid artery to cavernous sinus, of which internal carotid cavernous sinus fistula accounts for about 75%, and the most common cause is head and maxillofacial trauma (70%‐90%).^[3]^ There are also relatively rare reasons, such as transsphenoidal surgery, carotid artery angioplasty, and blood flow guide device insertion, which involve the cavernous segment of the carotid artery.^[4–6]^ Barrow et al classified TCCF into four types: A, B, C and D based on pathophysiology.^[5]^ The clinical manifestations of TCCT are mainly related to the structure of the cavernous sinus and the drainage vein. When TCCF occurs, arterial blood directly flows into the cavernous sinus, causing the pressure of the cavernous sinus to increase and the ophthalmic vein to expand, causing a series of ocular symptoms such as exophthalmos and conjunctival congestion and edema, subarachnoid hemorrhage may even occur.^[2,6]^

In the clinical diagnosis of this disease, DSA remains the gold standard to diagnose TCCF.^[7]^ Because the clinical ocular symptoms of this disease are obvious, in most cases, the patient will first seek medical advice from the ophthalmology department, and often the curative effect is poor. When the disease is delayed, there may even be serious consequences such as blindness. Therefore, it is very necessary to perform cerebrovascular examination, such as MRA, computed tomography angiography (CTA), etc. After considering TCCF through relevant examinations, further DSA examination is required to make a clear diagnosis. Because of the particularity of the clinical symptoms of this disease, it is often considered that it may be caused by this disease only after consultation with the neurology department. It is very easy to misdiagnose and miss diagnosis in clinical practice, so we must be highly vigilant.

At present, endovascular interventional therapy is the preferred treatment for this disease, and more than 90% of TCCF patients can be successfully cured by endovascular interventional therapy,^[8]^ which mainly includes the use of detachable balloon occlusion fistula, spring coil, Onyx glue, coated stent occlusion fistula or internal carotid artery. In this case, a case of TCCF was treated by stent implantation, which is expected to provide help for clinical identification and treatment of this disease and reduce the occurrence of missed diagnosis, misdiagnosis and adverse events of this disease.

## 4. Conclusion

TCCF is easy to be misdiagnosed clinically. When similar symptoms are encountered clinically, this disease must not be ignored. When suffering from this disease, better clinical efficacy can often be achieved through interventional treatment.

## Author contributions

**Conceptualization:** Xiaoming Shen.

**Data curation:** Niu Miao.

**Formal analysis:** Guofang Yang.

**Investigation:** Yangyang Liu.

**Methodology:** Dongyi Yang, Mingqin Zhang.

**Writing – original draft:** Jiabin Wang.

**Writing – review &amp; editing:** Tao Wu.

## References

[1] Liang J Xie X Sun Y. Bilateral carotid cavernous fistula after trauma: a case report and literature review. Chin Neurosurg J. 2021;7:46. 34736536 10.1186/s41016-021-00265-x PMC8567609

[2] Pülhorn H Chandran A Nahser H. Case report: traumatic carotid-cavernous fistula. J Trauma Nurs. 2016;23:42–4. 26745539 10.1097/JTN.0000000000000174

[3] Hamedani H Hellmann D Boyce W. Traumatic carotid-cavernous fistula: a case report. Radiol Case Rep. 2022;17:1955–8. 35432674 10.1016/j.radcr.2022.02.065 PMC9010516

[4] Iancu D Lum C Ahmed ME. Flow diversion in the treatment of carotid injury and carotid-cavernous fistula after transsphenoidal surgery. Interv Neuroradiol. 2015;21:346–50. 26015526 10.1177/1591019915582367 PMC4757274

[5] Wendl CM Henkes H Martinez Moreno R. Direct carotid cavernous sinus fistulae: vessel reconstruction using flow-diverting implants. Clin Neuroradiol. 2017;27:493–501. 27129454 10.1007/s00062-016-0511-6 PMC5719129

[6] Ting W Richard SA Changwei Z. Concomitant occurrence of clinoid and cavernous segment aneurysms complicated with carotid cavernous fistula: a case report. Medicine (Baltim). 2019;98:e18184. 10.1097/MD.0000000000018184 PMC6890289 31770272

[7] Nakae R Nagaishi M Takano I. Transvenous coil embolization for the treatment of carotid cavernous fistula after pipeline placement: a case report. J Stroke Cerebrovasc Dis. 2018;27:e65–9. 29174523 10.1016/j.jstrokecerebrovasdis.2017.10.023

[8] Chaudhry IA Elkhamry SM Al-Rashed W. Carotid cavernous fistula: ophthalmological implications. Middle East Afr J Ophthalmol. 2009;16:57–63. 20142962 10.4103/0974-9233.53862 PMC2813585

